# Baltimore College of Dental Surgery

**Published:** 1847-03

**Authors:** 


### Miscellaneous Notices.

*Baltimore College of Dental Surgery.*—The *annual commencement*, for conferring the degree of Doctor of Dental Surgery, was held in the Hall of the College Building, on the evenings of the 15th and 16th of February, ult.

On the first evening, the mechanical specimens of the candidates were exhibited to the audience, showing their skill and proficiency in the practical part of their profession—while, at the same time, suitable comments were made on the same, by Profrs. Westcott and Harris.

Prof. Bond then delivered an address; after which the exercises closed by a few pertinent remarks to the students, from Dr. Parmly, of New York, the venerable chairman of the *awarding committee.*

On the second evening, the exercises opened with music; then the candidates were called up, who, on taking their stand in the order of their names, the Dean of the Faculty stated that these gentlemen, having furnished the required testimonials of their several qualifications, now publicly presented themselves for the highest honors of the College; and, after reading in Latin the authority for conferring such honors, the President of the Faculty, Dr. C. A. Harris, then proceeded to confer the degree of Doctor of Dental Surgery, by handing to each one a diploma.

Music again enlivening the scene—Dr. Parmly came forward, and read the report of the awarding committee, consisting of Dr. Parmly, of New York, Dr. Roper, of Philadelphia, and Dr. Noyes, of Baltimore. Dr. Parmly, as chairman of this committee, stated the task that had been assigned them was, to award to the graduating student showing the highest proficiency, a set of dental instruments manufactured by Mr. Arnold of

Baltimore, and put up in the most workmanlike manner, in a very beautiful mounted rose-wood case, the whole costing not less than $50; and furnished by the Faculty, as a stimulus to, as well as a reward of merit.

The chairman announced that the committee had made a faithful investigation of the several specimens for the prize, and that, without any intercourse with any of the Faculty, had decided that Mr. Wayland, of Pa., was the successful competitor, and duly entitled to the award.

But, the chairman continued, notwithstanding this decision of the committee, as forming part of their official duty, they did not wish, by any means, to be understood as in the least disparaging the claims of the rest of the graduates; but, on the contrary, that they were each entitled to equal praise, equal confidence, and equal patronage from the public, as will be seen from the following report:

The committee appointed by the Faculty of the Baltimore College of Dental Surgery, to examine into the respective merits of the class of graduates, in order to award a token of approbation for the highest attainment in surgical and mechanical dentistry, acquired in that institution, beg leave most respectfully to report:

That, inasmuch as the award is intended solely as an expression of high approbation by the College, of proficiency and skill acquired while under its instruction—the committee, in justice to the institution and to the students respectively, have come to the decision that Mr. John Wayland has made such proficiency under the tuition of the College alone, independent of all other acquirements, as to entitle him to this mark of its favor.

In making this award, your committee, in justice to others, must also say, that the specimens they have produced in mechanical dentistry, and the operations they have exhibited in dental surgery, entitle them to equal praise—equal confidence and equal consideration. But, from the fact that the knowledge which they severally possess had been obtained partly from other sources, and partly from professional experience previous to their entering the institution, the committee could not, in justice, rank them as competitors for a prize, offered by the College for the highest excellence attained by an alumnus more especially its own. These gentlemen, therefore, are equally entitled, from their merits, to the same rank and estimation that this expression of approbation confers upon the recipient of it. They are upon a parity as to professional attainment, and they possess a skill in the operative department of the art, which, if carried out in their labors, will enable them to reach the very highest walks in practice, as surgeon dentists.

The committee would take leave to state, that no conversation was held with the professors, or with any one else, in relation to the graduates; nor was there any conversation between the members of the committee, as to the individual acquirements of the students; each member was simply requested to write the name of the candidate he considered the most successful, without the privacy of his colleagues. Your committee would finally state, that the same name was written by each of its members respectively.

E. PARMLY,<br>LEWIS ROPER,<br>E. NOYES, } *Examining and Awarding Committee.*

BALTIMORE, *16th Feb'y*, 1847.

The doctor then proceeded to address the students, recounting, in a poetical description, the history of his early life, with its numerous trials and difficulties, and his ultimate complete success, as a stimulus for them not only to hope but to persevere—to be faithful—to be honest—to act always as becoming workmen worthy of their profession, and of the profession to be worthy and proud of them—and there would be no danger of the result.

The doctor now taking his seat, and after having music again from the band, Prof. Westcott came forward, and pronounced the valedictory to the graduates, giving them wholesome advice, with the farewell and best wishes of the faculty for their future happiness and success.

The audience were now dismissed—music enlivening the dispersion.

*Names of Graduates, with their Residence and Subject of Thesis.*

J. N. BAIRD, Virginia; Mechanical Dentistry.

JNO. C. BAGBY, Virginia; The Deleterious Effects of Salivary Calculus.

W. E. MURPHY, S. Carolina; Diseases of the Maxillary Sinus.

G. LUCY, Alabama; Constitutional Effects of Diseased Teeth.

THOS. PALMER, Massachusetts; Indications for the Extraction of Teeth.

JNO. WAYLAND, Pennsylvania; On the Importance of the Preservation of the Teeth.

W. H. DAVIDSON, Tennessee; Principles and Practice of Inserting Artificial Teeth.

JNO. D. WEMPLE, N. Carolina; Observations on First Dentition in Producing Disease.

DR. McCULLOCH, Dublin; Science in connection with Natural History, and in connection with Dental Science.

DR. A. T. CONE, Massachusetts; On Inflammation of the Gums.

